# Assessing the impact of mitigation measures on cattle movement patterns in Austria: bluetongue virus outbreaks as a case study

**DOI:** 10.3389/fvets.2026.1800269

**Published:** 2026-04-27

**Authors:** Reinhard Fuchs, Beate Conrady, Ian Kopacka, Oliver Alber, Manfred Füllsack, Klemens Fuchs

**Affiliations:** 1 Department for Data, Statistics and Risk Assessment, Austrian Agency for Health and Food Safety, Graz, Austria; 2 Department of Environmental Systems Sciences, University of Graz, Graz, Austria; 3 Department of Veterinary and Animal Sciences, University of Copenhagen, Frederiksberg, Denmark

**Keywords:** bluetongue virus, control measures, livestock movements, network analysis, time series analysis

## Abstract

Outbreaks of infectious animal diseases pose significant challenges, including economic losses, animal welfare concerns, and disruptions to livestock production systems. Livestock movement is a critical transmission route during such outbreaks, and movement restrictions are commonly employed as spread mitigation measures. Understanding how such regulatory interventions affect livestock movement networks is essential for planning and implementing contingency measures and designing effective surveillance strategies. However, few studies have analyzed the interactions between network properties and regulatory perturbations during animal disease outbreaks. In this study, we assess the effects of two bluetongue outbreaks and their associated mitigation measures on the Austrian cattle movement network between 2003 and 2022. Using social network analysis and time series methods, we compare network properties using monthly cattle movement networks before, during, and after the outbreaks to identify any unexpected changes and assess network stability and variability over time. We found that the introduction of restriction zones led to temporary disruptions in network connectivity and reduced movement volumes, particularly when highly interconnected regions were separated by restriction zone boundaries. Many observed network metrics fell outside their predicted intervals shortly after the introduction of restriction zones. Subsequent measures that facilitated cattle movements, such as the establishment of a single nationwide restriction zone, vaccination of animals and vector-free periods, returned network indicators to expected levels, highlighting a notable degree of system resilience. Our findings demonstrate how network and time series analysis can be used to retrospectively evaluate the impact of regulatory interventions on livestock movement systems. The methodology presented here can assist veterinary authorities in evaluating disease outbreaks, understanding the impact of intervention measures on livestock trade, and planning future mitigation strategies. Ultimately, this approach supports veterinary authorities in effectively managing disease containment and minimizing disruption to livestock trade.

## 1 Introduction

Infectious diseases in livestock present a major global challenge, resulting in considerable economic losses, potential social consequences, and implications for animal welfare. The transmission of these diseases occurs through multiple pathways, with the movement of live animals playing a pivotal role. Many studies have examined the role of trade networks and their structures in either facilitating or impeding the spread of livestock diseases (1–17).

Having accurate information about the topology of movement networks of animals and their products can help in risk assessment and allows for the design of targeted, effective, and efficient surveillance and control strategies. Numerous studies have demonstrated the value of network-based approaches across a wide range of countries and production systems, including Germany (3, 18–20), Denmark (8, 21, 22), Sweden (23), Austria (24, 25), Switzerland (16, 26), France (11, 27), Slovenia (28), Italy (2), Spain (9), North Macedonia (29), the United Kingdom (30, 31), Ireland (14), Uruguay (32), and Brazil (33).

Insights into the epidemic dynamics of an outbreak can be gained by organizing the trade connections of a movement network over time and considering the sequential causality of these links. This approach, known as temporal network analysis, enables a more precise understanding of how infection pathways evolve over time, considering both the timing and sequence of contacts within trade networks (21, 23, 24, 34–39). When analyzing temporal networks, it is crucial to consider an appropriate temporal granularity for the analysis. While daily time frames are suitable for capturing short-term variations in the transmission dynamics of rapidly spreading diseases such as foot-and-mouth disease, less contagious diseases such as bluetongue (BT) are better suited to a coarser resolution in order to accurately detect significant changes (21, 22, 32).

During an outbreak, disease-specific movement restrictions are often imposed to mitigate the associated risks (40). Thus, not only does the movement network affect the spread of animal diseases, but conversely an animal disease outbreak can also have an impact on the movement network. So far, only few studies have conducted a network analysis assessing the effects of animal disease spread in combination with regulatory perturbations on animal movement such as in the study by Vernon and Keeling (41), Dutta et al. (27), and Conrady et al. (22). From a methodological perspective, analyzing such perturbations provides insight into how external interventions reshape movement networks. To evaluate and enhance the effectiveness of these intervention measures, detailed knowledge of outbreak-related interventions, regulatory frameworks governing animal movement, and the dynamic patterns of such measures is essential (35). According to Dubé et al. (7) a retrospective assessment of animal movements following an animal disease can provide useful information about improvements for future intervention measures.

In this study, we analyze the effects of large-scale movement restriction zones on the cattle movement network within Austria, using bluetongue as a case study to demonstrate the application of network and time-series analysis, rather than focusing on bluetongue-specific disease dynamics. Specifically, the goals are: (i) to compare network properties before, during, and after outbreaks of bluetongue virus serotypes 8 (BTV-8) and 4 (BTV-4) based on cattle movement data from 2003 to 2022, and (ii) to identify unexpected patterns and assess the stability and variability of the networks over time. Our analysis explicitly focuses on effects at the network level and not on changes at the node level. Although the analytical framework presented here is illustrated using bluetongue-related mitigation measures, it is not disease-specific and can be transferred to other infectious diseases and livestock production systems. The methodology presented here can assist veterinary authorities in evaluating disease outbreaks and the impact of interventions on livestock movements. This will support veterinary public authorities in planning future mitigation strategies.

## 2 Materials and methods

### 2.1 Bluetongue outbreaks and associated mitigation measures in Austria (2008–2019)

Bluetongue is a viral disease transmitted by biting midges, affecting ruminants such as sheep and cattle. It causes fever, swelling, and hemorrhages, leading to severe health issues, suffering, and sometimes death in affected animals. To date, 36 different serotypes have been identified, exhibiting clear differences in virulence.^1^

The first outbreaks of BTV-8 in Europe were reported in 2006 (42, 43). As a result, many European countries established surveillance and intervention measures, such as vaccination and/or movement restrictions. Directive 2000/75/EC foresees three hierarchical movement restriction zones in the case of a BT outbreak: an infected zone with a radius of 20 km around the infected holding, a protection zone with a radius of 100 km around the infected holding and a surveillance zone with a radius of a further 50 km beyond the protection zone. The protection and surveillance zones together are known as the restricted zone [Commission regulation (EC) 1266/2007]. Cattle and other susceptible animal species may only be moved out of a restricted zone under specific conditions listed in Annex 3 of Commission regulation (EC) 1266/2007: the animals are vaccinated, the animals are moved during a seasonally vector-free period or the animals are serological tested. Further details and conditions can be found in the text of the regulation.

**Figure 1:**
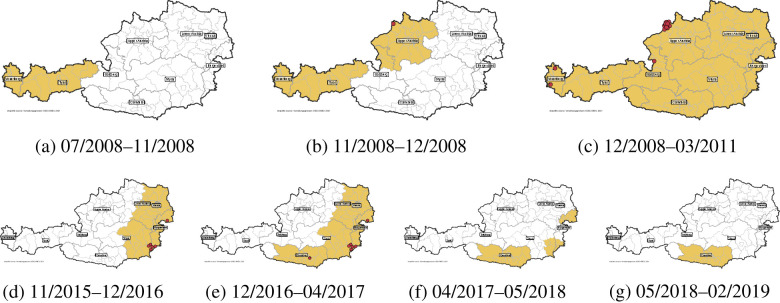
Bluetongue (BT) restriction zones in Austria 2008–2019. The top row shows the restriction zones of the BTV-8 outbreak in 2008/2009; the bottom row shows the restriction zones for the BTV-4 outbreak in 2015/2016. Cases are marked as red dots. Corresponding regulations “Blauzungenkrankheit-Bekmpfungsverordnung” (69) for figure **(a)** BGBl. II Nr. 267/2008, **(b)** BGBl. II Nr. 396/2008, **(c)** BGBl. II Nr. 96/2011, **(d)** BGBl. II Nr. 87/2015, **(e)** BGBl. II Nr. 428/2016, **(f)** BGBl. II Nr. 108/2017, **(g)** BGBl. II Nr. 95/2018, BGBl. II Nr. 37/2019. The referenced version of the national regulation can be found under https://www.ris.bka.gv.at/

The first small-scale surveillance zones in Austria were established in the western region at the end of 2007 in response to German cases near the Austrian border (44); these zones were lifted in spring of 2008 (BGBl. II Nr. 148/2008). In November 2008, BTV-8 was first detected in Austria (45). However, a mandatory nationwide vaccination campaign for all ruminants had been initiated in Austria four months prior, in response to the emergence of BT cases in Germany near the Austrian border. This campaign initially focused on the federal states of Vorarlberg and Tyrol (excluding East Tyrol; see Figure 1a). After detection of the first case in Austria, the vaccination zones were extended to the federal state of Upper Austria and parts of the state Salzburg in November 2008 (see Figure 1b). In December 2008, the entire country was designated as a single vaccination zone and subsequently classified as a single restriction zone after a second BTV-8 case was identified in Austria (see Figure 1c) (45). This was done to lift restrictions on animal movement between restricted and non-restricted areas, thereby facilitating the national trade in animals (46). In total, 28 BTV-8-positive cattle were identified across 14 farms in Austria between the years 2008 and 2009 (47). In mid-2009, the compulsory vaccination in Austria was replaced by a voluntary vaccination scheme.

The EU granted Austria BTV-free status in March 2011. However, this status was revoked in November 2015 after the detection of BTV-4 in Austrian cattle. Between 2015 and 2016, BTV-4 was detected in 10 animals across seven agricultural holdings in the federal states of Styria, Burgenland, and Carinthia (46). During this period, new restriction zones were established in parts of Lower Austria and Styria, and throughout the entire federal states of Carinthia, Vienna and Burgenland (see Figures 1d–g). The last restriction zones were lifted in February 2019. Further details on previously and currently implemented mitigation measures, including established vector-monitoring programs for BTV in Austria, can be found elsewhere^2^ (46– 48).

### 2.2 Cattle movement data

In Austria, all cattle movements (movements between agricultural holdings, to slaughter, to/from pastures, imports, exports) must be reported daily within 7 days with the exception of movements to/from pastures which are to be reported daily within 14 days. The reporting process underlies the Austrian legislation set forth in the “Rinderkennzeichnungs-Verordnung 2008” [BGBl. II Nr. 201/2008 (49), as amended by BGBl. II Nr. 66/2010 (50)]. All cattle movement data are stored in the Austrian cattle database hosted by Agrarmarkt Austria (AMA). From there, the data are imported into the official consumer health information system [Verbrauchergesundheitsinformationssystem (VIS)], which is owned by the Austrian Federal Ministry of Labor, Social Affairs, Health, Care and Consumer Protection and maintained by Statistics Austria.

In this study, we considered cattle movement data between January 1, 2003 and December 31, 2022 (20 years). Data from 2003 to 2009 were provided directly by AMA, data from 2010 on were extracted from the VIS database. It should be noted that the records prior to 2010 do not contain any movements to or from pastures, as different reporting obligations were in place during this period. They also do not include movements of cattle between holdings of the same owner. After 2010, these movements are included in the data if the holdings are located in different municipalities.

The analyzed data contained all cattle movements within Austria during the study period, with the exception of movements of cattle to slaughter, as movements of animals from a holding located in a restricted zone for immediate slaughter are exempted from the exit ban under conditions stated in Article 8 of the Commission regulation (EC) 1266/2007. Movements from and to other countries were not considered in this study in order to keep the focus on the network structure within Austria.

Movements are recorded using a single notification approach, whereby each holding only reports movement into or out of its holding, but not the other farm involved in the movement. Due to this system, the in- and out-movement data for each animal had to be matched. The resulting dataset comprised 16,234,245 domestic movements of cattle; 97 % of these could be unambiguously matched. The remaining 3 % were attributable to the following issues: multiple reported locations of the same animal on a single day (1.0 %); discrepancies in reporting dates—which were corrected for time differences of one to three days (1.3 %); and missing data, for which an algorithm assigned the most probable source or target holding when possible (0.3 %), while the unmatched remaining issues (0.4 %) were excluded from the dataset. After the matching and cleaning, individual cattle movements were aggregated into batches. A batch is defined as a group of animals moved from the same source to the same target node on the same day. The number of animals in each batch was recorded in an additional data field.

As an initial step, we analyzed the volume of cattle movements both within and between federal states. A high volume of animals moving between states likely contributes to the network's sensitivity to movement restrictions, as these restriction zones often follow state or district borders.

### 2.3 Network analysis of cattle movements

Movements of animals between holdings can be considered as a network and analyzed using network analysis methods. In this context, holdings represent the nodes of the network, and cattle movements between holdings represent the edges. As animal movements are directional (i.e., there is a direction of movement from node A to node B), the resulting graph can be considered a directed network. Networks are typically characterized using various network measures. These measures help to quantify and describe the structure, dynamics, and interactions within the network. The following paragraphs provide an overview of several measures that are widely used in veterinary epidemiology (e.g. 7, 22, 24, 27, 38, 51, 52).

#### 2.3.1 Degree

The degree of a node represents the number of edges (or connections) it has to other nodes in the network. In directed networks, degree is further divided into two types: in-degree, which is the number of incoming edges, and out-degree, which is the number of outgoing edges. A node with a high degree is often referred to as a hub, indicating that it is highly connected and plays a critical role within the network. In the context of animal movement networks, the presence of highly connected nodes can significantly accelerate the spread of a disease (53). The total degree, in- and out-degree were calculated for the unweighted networks. The medians of the mean node-level values of the monthly networks are shown in Supplementary Tables S1, S2.

#### 2.3.2 Average path length

The average path length measures the average number of edges in the shortest paths between all pairs of nodes in a network (53). It indicates how interconnected the nodes are (54). In animal movement networks, a shorter average path length suggests that animals (and diseases) can quickly move between holdings, affecting the time it takes for a disease to spread through the network.

#### 2.3.3 Diameter

The diameter of a network is the maximum value of all shortest paths between any pairs of nodes (53). It is used to provide an overview of the possible velocity of an outbreak and in general measures network cohesion (51).

#### 2.3.4 Degree correlation

Degree correlation refers to the preference for a network's nodes to attach to others that have a similar (or dissimilar) number of connections; it is therefore a special case of assortativity mixing (see below). A positive degree correlation indicates that high-degree nodes tend to be connected to other high-degree nodes, while a negative correlation implies that high-degree nodes tend to be connected to low-degree nodes (53, 55).

#### 2.3.5 Connected components

Connected components are groups of nodes that are (directly or indirectly) connected to each other by at least one edge. A strongly connected component refers to a group of nodes in a directed network where each node can be reached along the network from every other node. In contrast, a weakly connected component refers to a group of nodes that are connected not taking the direction of the edges into account. The giant strongly connected component (GSCC) and the giant weakly connected component (GWCC) are the largest respective connected components in a network (53).

#### 2.3.6 Assortativity/mixing patterns

Assortativity is the tendency of nodes to connect to similar nodes (53, 55). We analyzed assortativity in two ways. First, we examined the extent to which nodes tend to have edges with other nodes within the same district. Second, we investigated the extent to which nodes have edges with nodes within the same federal state.

#### 2.3.7 Relative reachability

Relative reachability indicates the average proportion of nodes that can be reached in the network from any random node considering the temporal sequence of the links. In our case, this means that a link is only active on the specific day of the movement. The proportion is calculated based on the active nodes in the respective network. In the veterinary epidemiological context, this measure indicates how many holdings can be directly or indirectly affected on average in the event of an animal disease outbreak (52).

#### 2.3.8 Loyalty

The temporal stability of networks can be quantified using its “loyalty,” which measures the proportion of connected node-pairings that remain connected in the network over a set of discrete time windows (56). The loyalty measure was calculated for time windows of calendar years and for undirected networks.

All of the above metrics describe the network at a global level, except for degree and relative reachability, which are node-level metrics. To enable comparison at the network level, mean values of these two metrics were also calculated. Moreover, the results of the network metrics in Table 1 and Supplementary Tables S1, S2 are presented on an annual basis, with each year represented by the median of its corresponding monthly values.

**Table 1:** Movements within restricted area, from free to restricted area and from restricted area to free area for three settings.

**Start**	**End**	**Total**	**Within area (%)**	**To area (%)**	**From area (%)**
2006-07-30	2006-08-26	37,380	9.2	3.0	0.2
2007-07-30	2007-08-26	37,124	9.1	2.8	0.5
2008-07-30	2008-08-26	38,152	8.1	2.3	0.5
2009-07-30	2009-08-26	36,732	7.6	2.2	1.4
2010-07-30	2010-08-26	44,773	6.5	1.6	1.1
2006-11-19	2006-12-16	58,914	43.7	6.7	5.7
2007-11-19	2007-12-16	58,130	46.4	7.1	4.9
2008-11-19	2008-12-16	60,184	43.2	6.4	4.0
2009-11-19	2009-12-16	60,742	44.7	6.9	3.7
2010-11-19	2010-12-16	70,602	40.3	7.8	4.1
2013-11-20	2013-12-17	70,424	14.0	5.6	4.1
2014-11-20	2014-12-17	69,558	13.9	5.7	4.2
2015-11-20	2015-12-17	70,477	14.0	3.4	0.8
2016-11-20	2016-12-17	71,719	14.3	3.9	1.8
2017-11-20	2017-12-17	74,215	11.9	5.6	3.4

In the first five rows, the considered area is the restriction zone implemented in 2008-07-30, see Figure 1a; in the middle five rows the considered area is the restriction zone implemented in 2008-11-19, see Figure 1b and the in the bottom five rows the considered area is the restriction zone implemented in 2015-11-20, see Figure 1d. Within each setting, the areas are fixed.

### 2.4 Time series analysis

For the time series analysis of the network measures, the cattle movement network was divided into monthly sub-networks. In total, 240 monthly cattle movement networks were created over the 20-year period. For each of these monthly networks, various network measures outlined in Section 2.3 were computed, resulting in a time series for each measure.

To examine the temporal evolution of these static monthly networks over the study period, time series analysis models were employed. A widely utilized and effective approach are Seasonal Autoregressive Integrated Moving Average (SARIMA) models. The basis of a SARIMA model is a stationary stochastic process with a linear structure of lagged observation and error terms. A comprehensive overview of SARIMA models can be found in classic time series texts, such as Brockwell and Davis (57) and Box and Jenkins (58).

The primary reasons for employing SARIMA models are their ability to model seasonality, which is defined in the model structure in form of seasonally lagged terms, as well as their predictive and statistical properties. Even though SARIMA models are most effective for short-term predictions, long-term predictions are useful in this context for comparing time periods of interest for actual developments and the SARIMA predictions.

For each network measure time series, the coefficients of the SARIMA model were estimated by inspection of the autocorrelation function and maximum likelihood methods. Suitable models were selected based on the statistical significance of the estimated coefficients as well as likelihood-based information criteria, in particular AIC. Conventional model diagnostics checks, such as deviation from white noise, were performed for each series. As there are several fitted SARIMA models, the respective estimated coefficients and information criteria are not included and discussed in detail but are available on request. The fitted models were then used to calculate predictions and prediction intervals for future observations.

The primary objective of the time series analysis was to predict values of network measures for a given period based on data from a preceding period. We then compared these predictions, specifically the bounds of the prediction intervals, with the actually observed values. Predictions that fall outside the prediction interval can be regarded as unusually large deviations from the estimated model. This approach allowed us to analyze the time required for the movement network to revert to its original state following an outbreak, to evaluate whether such reversion occurred at all, and to identify unexpected patterns and assess the stability and variability of the networks over time.

The data were analyzed across three distinct time periods for BTV-8: from 2003 until mid 2008 (referred to as before the BTV-8 outbreak), between mid 2008 and mid 2011 (during the BTV-8 outbreak) and from mid 2011 until 2014 (after the BTV-8 outbreak). For BTV-4, we analyzed the periods between 2010 and 2015 (before the BTV-4 outbreak), from 2016 until February of 2019 (during the BTV-4 outbreak) and between February 2019 and 2022 (after the BTV-4 outbreak). Data from the periods before each outbreak was used to train the SARIMA models. Predictions for the periods during the outbreaks were generated using the models to compare with the actually observed data. The corresponding time periods following each outbreak were then presented to visualize the subsequent development.

### 2.5 Software

For all analyses, we used the programming language R (v4.4.3; 59). Data cleaning, processing, and plotting utilized the “tidyverse” packages (60) and for chord diagrams the “circlize” package (61) was used. The networks were constructed and analyzed using the packages “igraph” (62) and “EpiContactTrace” (52).

## 3 Results

### 3.1 Overview and descriptive analysis of the network

When comparing network properties over our whole study period, we observed notable changes in the structure of the Austrian cattle sector. Table 2 provides a summary of the analyzed data. Over the 20 years covered by this study, the number of cattle holdings decreased by 40.2 %, from 87,899 in 2003 to 52,568 in 2022 (63). This decline was accompanied by an 8 % reduction in Austria's total cattle population over the same period, which is reflected in an increase in the average farm size from 23 to 35.4 animals per farm. Along with the reduction in the number of cattle farms, the number of active network nodes decreased by 16.5 % while showing a slight increase in the proportion of active nodes. Despite the decline of the cattle population, the number of animals moved between farms increased by 52.3 %, from 46,310 in 2003 to 70,541 in 2022 per month on average.

**Table 2:** Summary metrics of the Austrian cattle movement network from 2003 to 2022.

Metrics	2003	2004	2005	2006	2007	2008	2009	2010	2011	2012
No. cattle	2,021,949	2,003,601	2,010,434	2,002,827	2,000,196	1,997,210	2,026,260	2,013,277	1,976,542	1,955,618
No. holdings	87,899	85,292	82,922	80,179	77,482	75,209	73,476	71,578	69,604	67,655
No. active nodes	27,054	26,639	26,690	26,581	25,801	26,139	24,995	25,424	26,575	26,366
No. batches	29,595	30,209	30,779	31,008	30,012	31,935	28,945	30,528	33,437	32,748
No. cattles moved	46,310	49,133	50,014	53,694	55,295	56,633	54,220	57,769	67,700	67,115
No. source-nodes	19,786	19,441	19,719	19,630	18,989	19,340	18,391	19,015	20,137	20,001
No. target-nodes	10,790	11,052	10,765	10,765	10,610	10,702	10,311	10,294	10,536	10,488
Average path length	7.50	6.94	7.06	6.94	6.82	6.26	6.67	6.29	6.25	6.08
Diameter	22	22	21	20	21	19	20	21	19	20
Degree correlation	–0.5563	–0.5442	–0.5472	–0.5434	–0.5357	–0.5315	–0.5416	–0.5200	–0.5095	–0.5047
GWCC	0.6950	0.7211	0.7320	0.7515	0.7576	0.7673	0.7429	0.7779	0.8092	0.8065
GSCC	0.0345	0.0386	0.0363	0.0426	0.0458	0.0460	0.0434	0.0439	0.0477	0.0446
Assortativity District	0.4175	0.4196	0.4097	0.4042	0.4077	0.4033	0.4037	0.3928	0.3817	0.3825
Assortativity Federal State	0.7392	0.7516	0.7504	0.7559	0.7685	0.7577	0.7615	0.7635	0.7615	0.7699
Relative reachability [%]	0.3121	0.3684	0.4086	0.5037	0.5720	0.8411	0.7068	0.7572	0.7927	0.8235
**Metrics**	**2013**	**2014**	**2015**	**2016**	**2017**	**2018**	**2019**	**2020**	**2021**	**2022**
No. cattle	1,958,283	1,961,201	1,957,610	1,954,489	1,943,590	1,912,808	1,879,521	1,855,440	1,870,100	1,861,071
No. holdings	65,699	63,527	61,778	60,571	59,282	57,861	56,389	55,019	53,656	52,568
No. active nodes	25,728	25,527	25,331	24,832	24,557	24,346	23,717	22,322	22,470	22,579
No. batches	31,553	32,870	32,254	31,852	32,418	31,269	31,849	28,752	29,035	29,888
No. cattles moved	62,763	66,696	67,412	69,528	69,532	71,079	70,735	64,871	66,499	70,541
No. source-nodes	19,145	19,159	19,145	19,095	18,707	18,430	18,125	16,821	16,959	16,873
No. target-nodes	10,543	10,428	10,331	9,938	10,162	9,963	9,359	9,140	9,425	9,375
Average path length	6.13	6.43	6.37	6.64	6.23	6.29	6.19	6.63	6.46	6.37
Diameter	20	20	20	22	20	20	19	20	21	20
Degree correlation	–0.5098	–0.5069	–0.4905	–0.4680	–0.4708	–0.4689	–0.4698	–0.4867	–0.4755	–0.4641
GWCC	0.7995	0.8061	0.8091	0.8202	0.8249	0.8230	0.8217	0.8104	0.8156	0.8199
GSCC	0.0472	0.0492	0.0510	0.0515	0.0485	0.0521	0.0484	0.0411	0.0408	0.0440
Assortativity District	0.3913	0.3910	0.3994	0.4004	0.3874	0.3846	0.3858	0.3926	0.3918	0.3959
Assortativity Federal State	0.7714	0.7784	0.8023	0.8122	0.8087	0.7975	0.7941	0.7947	0.7994	0.8043
Relative reachability [%]	0.8986	0.8169	0.8045	0.8628	0.8885	0.9138	1.0550	0.5922	0.7387	0.8815

Network measures were calculated for monthly networks and are shown as yearly medians, with the exception of the values for the number of (No.) cattle and No. holdings, which were derived from national statistics (Table 2.2.2.6 in (63)).

The number of moved batches remained relatively stable over the years. The average size of the batches, on the other hand, increased from 1.6 to 2.4 animals per batch. Throughout the entire study period, the number of source nodes (sellers) consistently exceeded the number of target nodes (buyers) in the network, highlighting an imbalance in the trade network.

Over the 20-year period, 3.4 % of cattle holdings maintained the same movement relationships in 2022 as in 2003. While year-to-year loyalty increased over the study period, rising from 17.7 % between 2003 and 2004 to 27.2 % between 2021 and 2022, this trend may also be influenced by the overall reduction in the number of cattle farms. The data for loyalty is presented in Supplementary Table S3.

**Figure 2:**
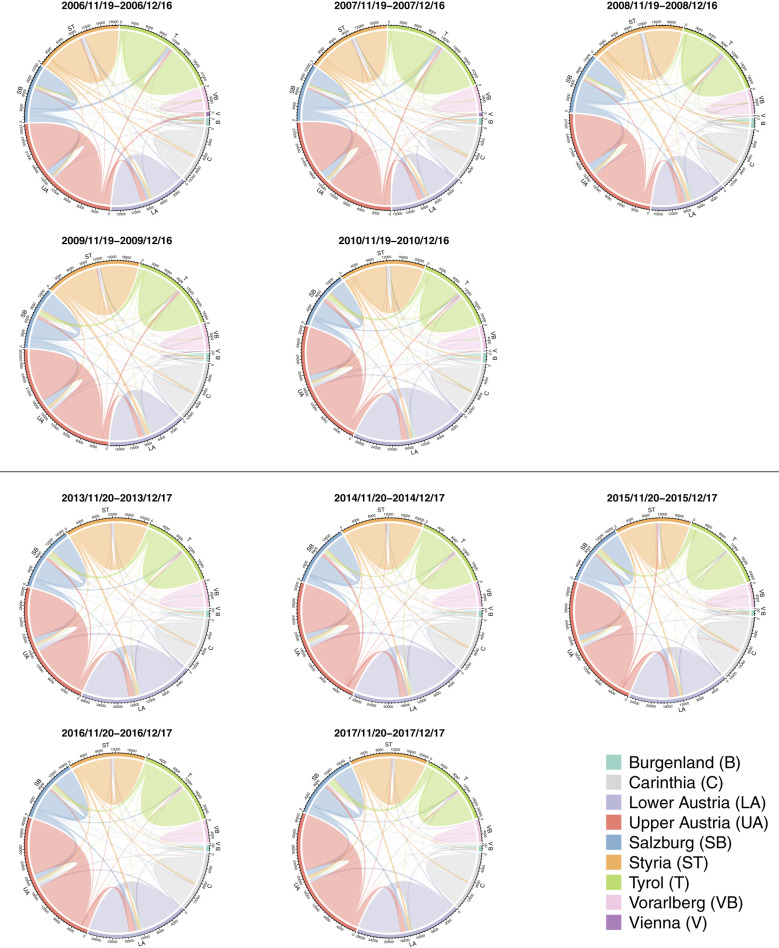
Cattle moved within a federal state and between federal states for a 4-week window in Nov./Dec. 2006, 2007, 2008, 2009, and 2010 **(top)** and Nov./Dec. 2013, 2014, 2015, 2016, and 2017 **(bottom)**. The sectors of the circle represent the federal states, while the arcs illustrate the movement of animals. The width of the arcs is proportional to the number of animals. The arcs are color-coded according to the state of origin, and their width corresponds to the volume of moved animals. Comparing the diagrams for 2008 and 2015 with those for the two preceding and two following years highlights the differences for federal states affected by restriction zones.

Figure 2 shows chord diagrams depicting the number of cattle moved within and between Austria's federal states for 4-week windows from around mid November to mid December for the years 2006–2010 and 2013–2017. These time windows were selected to examine the impact of the restriction zones implemented in November/December 2008 (as shown in Figure 1b) and 2015 (as shown in Figure 1d), compared to the two years preceding and following these periods. In general, the highest volume of within-state cattle movements occurred in Upper Austria, Lower Austria, Styria and Tyrol. Styria and Tyrol exhibited significant outgoing movements to other federal states, Lower Austria predominantly experienced incoming movements, while Upper Austria and Salzburg showed a balance of both incoming and outgoing movements. Carinthia and Vorarlberg had mainly within-state movements and very little out-/incoming movements. Burgenland and Vienna had very few cattle movements due to their low cattle population. The figure illustrates that during both periods when restrictions were implemented, the affected federal states experienced a decline in cattle movements. In 2008, Tyrol recorded the lowest number of cattle movements within the 2006–2010 period. Similarly, in 2015, both Styria and Lower Austria had the lowest number of cattle movements within the 2013–2017 period.

In addition, we analyzed the movement patterns for three distinct restriction zones implemented due to BTV outbreaks. Each restriction zone was analyzed over a 4-week period following its establishment, with comparisons made to the same 4-week periods one and two years prior, and one and two years after the implementation. Importantly, these comparisons are made within the same geographical regions, even though the restriction zones were not in place during the two years prior to their establishment. The first restriction zone, implemented in July 2008 and covering Vorarlberg and Tyrol, was due to BTV-8 cases in Germany. The second restriction zone, established in November 2008 and extending to Upper Austria and parts of Salzburg, was in response to the first BTV-8 cases in Austria. The third restriction zone, introduced in November 2015 in the east of Austria, was due to the first BTV-4 cases in Austria (as seen in Figure 1).

For each restriction zone, Table 1 records the total number of cattle movements within Austria during the first 4 weeks of the restriction zone. Additionally, it details the percentage of movements that occurred within the affected area, that entered the restricted area, and that exited the restricted area. These figures are then compared to the same 4-week periods one and two years before the establishment of the restriction zone, as well as one and two years after.

In the first restriction zone, the percentage of cattle movements out of the zone remained consistently low prior to and including the year of the restriction zone's establishment, at 0.5 %. However, following the establishment of the restriction zone, this percentage increased noticeably, rising to 1.4 %. For the second restriction zone, established in November 2008, which extended to cattle-dense regions in Upper Austria and Salzburg, there was a notable decrease in the proportion of cattle movements in the year of the restriction zone's establishment compared to the prior year. This decline was observed across all three categories: movements within the zone, movements from the zone, and movements into the zone. In the third restriction zone, introduced in November 2015, there was a significant reduction in cattle movements into the zone, dropping from 5.7 to 3.4 % when comparing the year of establishment to the prior year. Similarly, the percentage of movements leaving the zone decreased markedly, from 4.2 to 0.8 %. This highlights the impact of the imposed measures. Once these federal states were not isolated, the number of cattle movements stabilized.

### 3.2 Time series analysis of network properties

**Figure 3:**
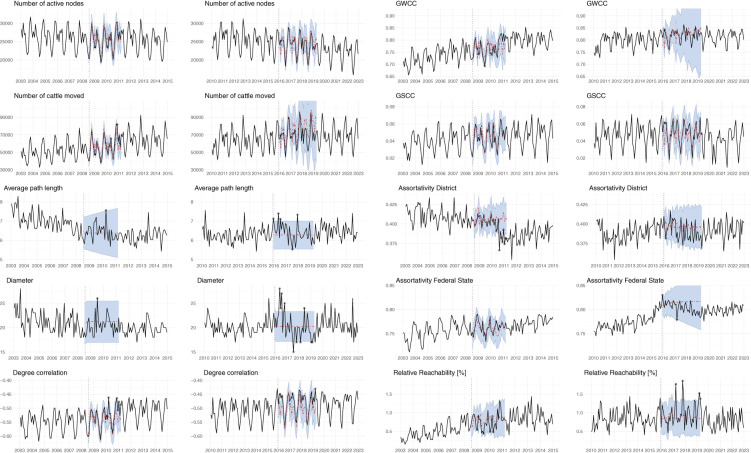
Analysis of cattle movements before, during, and after the BTV-8 outbreak (2003–2014; figures in columns 1 and 3) and the BTV-4 outbreak (2010–2022; figures in columns 2 and 4). Dotted vertical lines indicate the beginning of movement restriction zones. Black lines represent observed data, while red dash-dotted lines show SARIMA predictions with 95% prediction intervals in blue shading. Predictions are shown only during restriction-zone periods; asterisks indicate observations outside the prediction interval.

Table 2 summarizes the yearly median values of the monthly network measures, alongside annual cattle and holding counts from national statistics for the period 2003–2022. Figure 3 complements this overview by showing the full monthly time series of the network measures together with the prediction intervals generated by the SARIMA models. For BTV-8, shortly after the restriction zone was implemented in mid-2008, none of the observed values in Figure 3 fall outside the 95 %-prediction interval. Only toward the end of 2008/beginning of 2009, when the restriction zone was extended, is there a short period in which the observed values fall below the lower bound of the prediction interval for the measures active nodes, cattle moved, GWCC, GSCC, and relative reachability.

Starting in 2010, a new reporting obligation came into effect, resulting in an increased number of reported cattle movements. This change is reflected in certain network measures, such as cattle moved, degree correlation, and assortativity district. Consequently, any comparisons of analyses conducted before and after 2010 should be approached with caution.

For BTV-4, shortly after the restriction zone was implemented in November 2015, several network measures fall outside the prediction interval. This is evident for active nodes, cattle moved, GWCC, GSCC, and degree correlation. The time series for assortativity federal state shows a distinct change in pattern in 2015, with a slight decrease following the implementation of the BTV-4 restriction zone. The model predictions remain at the level observed at the end of 2015.

An exception is relative reachability, which significantly exceeds the prediction bounds in later years, with peaks in the first and last quarters of 2017, although it roughly remains at its original level for the remaining years. Degree correlation also deviates from its predicted values during the second time period, even though the actual values fall within the prediction bounds. For the first halves of 2016, 2017, 2018, and 2019, degree correlation shows much higher observed values than those predicted by the SARIMA model.

Almost all time series in Figure 3 exhibit strong seasonal patterns. The estimated seasonal coefficients of the SARIMA model are significantly different from zero for these series. The major exceptions are average path length and diameter for both time periods, where no significant autocorrelations were found. The observed series remain at a fixed level, showing no change in pattern after the implementation of restrictions. The seasonal series for active nodes, GWCC, GSCC, and degree correlation have highly significant seasonal autocorrelation coefficients with a period of 12 months.

It is evident that the SARIMA models represent most of the series fairly well. Highly seasonal series, such as active nodes, cattle moved, GWCC, and GSCC, can be modeled by SARIMA processes of relatively low order. It is remarkable that the long-term predictions are often fairly close to the observed values, regardless of the introduction of restriction zones. For other series, such as assortativity district for BTV-8 and assortativity federal state, there is a clear break in pattern and deviation from the assumptions of a SARIMA process. Given the suitability of these models, along with the often wide prediction intervals, observations that fall outside of the prediction bounds can be regarded as unexpected and should be analyzed further, especially if they occur shortly after implementation of a restriction zone.

Observations that break through the prediction bounds after implementation of the restriction zones tend to return quickly to the predicted bounds. Typically, the time series return within a few weeks, except for those with a clear change in pattern. Other series tend to break through more often for very short periods, such as relative reachability and degree correlation for BTV-4 and BTV-8. Degree correlation for BTV-4 appears to be at a much higher level than predicted by the model, even though the seasonal pattern remains the same.

## 4 Discussion

To the best of our knowledge, this study represents the first comprehensive network analysis of Austrian cattle movements over a period of 20 years. Specifically, we evaluated the effects of long-term and large-scale movement restrictions using two outbreaks of bluetongue disease as empirical case studies. Our aim was to identify unexpected movement patterns and to assess the stability and variability of the underlying network over time, rather than focusing on bluetongue-specific disease dynamics.

Our findings revealed substantial structural changes in the Austrian cattle sector over the study period. Specifically, we observed a decline in the cattle population, the number of cattle farms, and active nodes, despite an increase in the total number of cattle movements (Table 2). These trends are consistent with observations from other countries, such as the United Kingdom (64), France (27), Slovenia (28), or Denmark (22), where similar structural shifts have been reported. A key contributing factor to these changes appears to be the reduction in the number of cattle farms, which likely influenced the observed increase in movement loyalty (Supplementary Table S3). As fewer national trading partners remain available, cattle movements become more consistent over time. However, in addition to these economic and demographic shifts, the structural changes in the Austrian cattle movement network can be attributed to amendments in the legal framework in February 2010. This regulatory change introduced stricter reporting requirements for cattle movements (50). Specifically, it mandated the reporting of livestock movements to alpine pastures, as well as movements between farms owned by the same entity if they were located in different municipalities. This legislative amendment was reflected in a notable increase in the number of recorded cattle movements in 2010 and 2011, highlighting the influence of policy changes on the data availability, e.g. for modeling network dynamics or disease spread.

In Austria, there are considerable regional differences in the distribution of the cattle population and the intensity of cattle movements (Figure 2). These underlying spatial differences influenced the impact of movement restrictions on cattle movement patterns. At a finer temporal resolution, a reduction in cattle movements was observed when examining movements into and out of restriction zones (Table 1). Once the affected restriction zones were no longer isolated, cattle movement volumes returned to previous levels, indicating that the restrictions had a temporary but substantial impact on the movement network.

The time series analysis of the Austrian cattle network showed no unusual behavior immediately after the introduction of the BTV-8 restricted zones when only Tyrol and Vorarlberg were affected (as seen in columns 1 and 3 of Figure 3). This can be explained by the fact that these two federal states are not involved in much trade with other federal states (Table 1). It was only at the end of 2008, after Upper Austria and parts of Salzburg also became part of the restriction zone, that significant outliers in the time series of many network parameters became recognizable, even if they were only visible for a short period of time. This may indicate a temporary disruption in the movement network immediately following the extension of the zones. Fewer active nodes and moved cattle lead to a less connected network. Since relative reachability corresponds to the average proportion of holdings that can be reached, considering the temporal sequence of the links, a decline indicates a short-term reduction in the expected potential outbreak scale. Once the entire country was under a restriction zone, most network parameters appeared to stabilize again. Only the diameter, the giant weakly/strongly connected components, and the relative reachability still showed singular outliers during the year 2009. The legislative amendment to the reporting obligations introduced in 2010 is clearly reflected in the time series of the network measure assortativity district. This shows that, in addition to changes in movement behavior, changes in reporting behavior can also have an influence on network characteristics.

The situation for the BTV-4 outbreak and corresponding restriction zones in 2015 differed from the BTV-8 outbreak. The first case appeared very late in the year in the eastern part of Austria. Restriction zones were established immediately, and vaccination of animals was voluntary. The restriction zone extended across the cattle-dense federal states of Styria and Lower Austria. Most of the network measures in Figure 3 (columns 2 and 4) show outliers at the beginning of the outbreak. In particular, average path length and diameter show upward outliers during the year 2016, indicating that it takes a longer path to connect two holdings and that the network distance between the furthest holdings is greater. At the same time, the connected components showed no change at these time points. From this, it can be concluded that there would be a slowdown in terms of a possible disease spread velocity. After a short time, the network measures stabilized within predicted levels. Besides vaccination, another reason for this rapid stabilization was the declaration of a vector-free period during the winter months (65), which simplified the movement of cattle from the restriction zone to the non-restriction zone.

The network metrics reported in this study have clear practical relevance for outbreak control. Measures such as degree and betweenness centrality identify farms that occupy structurally influential positions within the cattle movement network and may contribute disproportionately to disease spread. These holdings can therefore be prioritized for targeted surveillance, vaccination, or temporary movement restrictions, potentially reducing epidemic spread more efficiently than uniform, population-wide measures. Monitoring overall network connectivity and comparing structures during and after outbreaks further helps assess system vulnerability and anticipate how future outbreaks may propagate. Overall, these findings support the use of network properties to inform surveillance strategies and control planning in livestock disease management.

Approaches that use predictions from time series models assume that the time series are well represented by the models used. SARIMA models make certain assumptions, in particular on autocorrelation structure, which should correspond to the sample autocorrelations of the time series to yield valid statements. Some series show very little autocorrelation, leading to overly simple model adjustments. SARIMA models are usually used for short-term predictions, whereas in this study, longer time periods were predicted. However, our results show a fairly good agreement between the predictions and the observed values, even for long prediction intervals. This suggests that the SARIMA model used was well suited for predicting and assessing the actual values after the implementation of the restriction zones. Overall, the SARIMA models showed that the number of active holdings and the number of moved animals exhibited good prediction accuracy and clearly captured seasonal trends and changes in network behavior. While the average path length and diameter measures were not highly accurate in terms of predictive performance, they nevertheless identified outliers very clearly. These outliers indicated meaningful changes in the underlying network structure. In contrast, the geographically based measures, assortativity by district and federal state, which reflect the extent to which holdings trade preferentially within or across administrative regions, showed comparatively poor predictive quality.

Comparing the network characteristics with those of other European countries shows that although some network indicators are similar, each network has its own individual characteristics. For instance, the average path length in Austria is similar to that in Denmark (22), France (27), or Italy (2), but shorter than in Switzerland (16) and longer than in Slovenia (28). The diameter of the Austrian cattle network is similar to the French cattle network (27), but is higher than in Slovenia (28). It is, therefore important to have detailed insights in the country-specific movement network in order to be able to react specifically in the event of an outbreak. Further, as shown by Duncan et al. (64), using the most recent data is recommended for network modeling to provide accurate results for decision making.

Austria is a mountainous country (66), and in alpine regions it is common practice to move cattle to alpine pastures during the summer months [see, for example, practices in Switzerland (16)]. Due to the unavailability of data for pasturing movements for the entire study period (data available since 2010), movements to and from pastures were excluded from the analysis. This exclusion could potentially skew the results. The Supplementary material contain analyses for the period from 2010 to 2022, which include pasture movements (see Supplementary Figure S1 and Supplementary Table S2). As demonstrated in other studies (16), the network measures differ considerably when movements to alpine pastures are included compared to when they are excluded, reflecting both seasonal (movements to pastures in May/June and from pastures in September) and geographical (mainly in alpine regions) differences compared to when such movements are excluded.

This paper focused on the movements of cattle, even though small ruminants also serve as hosts for BTV. We, however, assume that excluding the movements of sheep and goats, does not significantly impact the findings of this study. This assumption is based on the following considerations: (i) less than 18 % of farms in Austria house more than one species, in contrast to other countries like the UK, where 56 % of cattle farms also keep small ruminants and exhibit frequent movements between livestock species (67). (ii) During the study period in Austria, BT infections were reported exclusively in cattle farms, suggesting that small ruminants played a negligible role in disease transmission within the observed network. However, it is important to note that movement restrictions were also implemented for small ruminants in Austria.

Our analyses indicate that the regulatory measures introduced in response to the BT outbreaks are associated with changes in the network of cattle movements. However, disruptions to the movement network can also arise from other causes. For instance, other animal diseases, such as the Infectious bovine rhinotracheitis/infectious pustular vulvovaginitis (IBR/IPV) outbreak in Austria in 2015 (44), or significant events like the global COVID-19 mitigation measures implemented between 2020 and 2022 (25, 68), could have affected the calculated network properties in this study. These external confounding factors were not considered in this study.

The present study has several limitations. First, the introduction of new legal reporting obligations in 2010 caused a structural change in the dataset, influencing several network measures independently of their actual behavioral changes. As this modification affected only part of the BTV-8 outbreak period, the results up to that point can be interpreted without this limitation. Second, movements to and from alpine pastures were not available for the entire study period, and were therefore excluded from the main analyses. Results including these movements are provided in the Supplementary material. Third, we represented the dynamic cattle movement system as a series of monthly networks. Although this approach simplifies the true movement dynamics and may overestimate farm connectivity (32, 34), time-aggregated networks are widely used in veterinary epidemiology. A monthly time scale was considered epidemiologically appropriate for bluetongue virus (BTV), as diseases with lower contagiousness can be adequately studied using aggregated time scales. However, aggregating movements at this level may have led to an overestimation of certain network properties due to increased apparent connectivity. Finally, external events, such as other animal disease outbreaks or the COVID-19 pandemic related measures may have also affected the cattle movement network, but it can be assumed that the influence on the presented results is minor, as they did not coincide with the periods of the two BT outbreaks.

Further research could address several aspects not fully captured in this study. First, analyzing the network at alternative temporal resolutions, such as weekly or quarterly windows, may reveal additional short-term or long-term dynamics that are not visible at the monthly scale. Second, a detailed analysis of the cattle network including movements to and from alpine pastures for the years in which these data are available could help determine whether their exclusion influences the network structure or the interpretation of movement restrictions. Finally, including further relevant information, such as concurrent disease events, economic factors, or policy changes, could help to distinguish between true behavioral shifts from changes caused by external influences or reporting practices.

Despite its limitations, the study provides valuable insights for the international scientific and policymaking communities. The analysis and thorough understanding of the country-specific cattle movement network provides valuable information that is helpful in defining movement restrictions to minimize the risk of further animal disease spread, and in addition, the impact on livestock trade. The methods presented can be applied to future outbreaks of BT and similar diseases. This methodology framework can assist veterinary authorities in assessing disease outbreaks, understanding the impact of associated mitigation measures on livestock movement, and planning future strategies to contain the disease. By combining cattle movement, surveillance, and intervention data within a One Health framework, this study demonstrates how interdisciplinary approaches can improve understanding of disease spread and inform evidence-based policy.

## Data Availability

The raw data supporting the findings are not publicly available due to data protection regulations. However, data are available from the authors upon reasonable request and with the permission of the data owner, the Austrian Federal Ministry of Labour, Social Affairs, Health, Care, and Consumer Protection. The R code used to produce the results of this study is also available on request from the authors. Requests to access these datasets should be directed to reinhard.fuchs@ages.at.

## Footnotes

1
https://www.ages.at/en/human/disease/pathogens-from-a-to-z/bluetongue

2
https://www.verbrauchergesundheit.gv.at/tiere/tierkrankheiten/BTV.html
