# Supplementary material for: Assessing the impact of mitigation measures on cattle movement patterns in Austria: bluetongue virus outbreaks as a case study
Source: Front Vet Sci. 2026 Apr 27;13:1800269. doi: 10.3389/fvets.2026.1800269 (PMC13161693; doi:10.3389/fvets.2026.1800269)
Supplement: Supplementary file 1 [file Data_Sheet_1.PDF]

Supplementary Material

1 SUPPLEMENTARY DATA

1.1 Figures

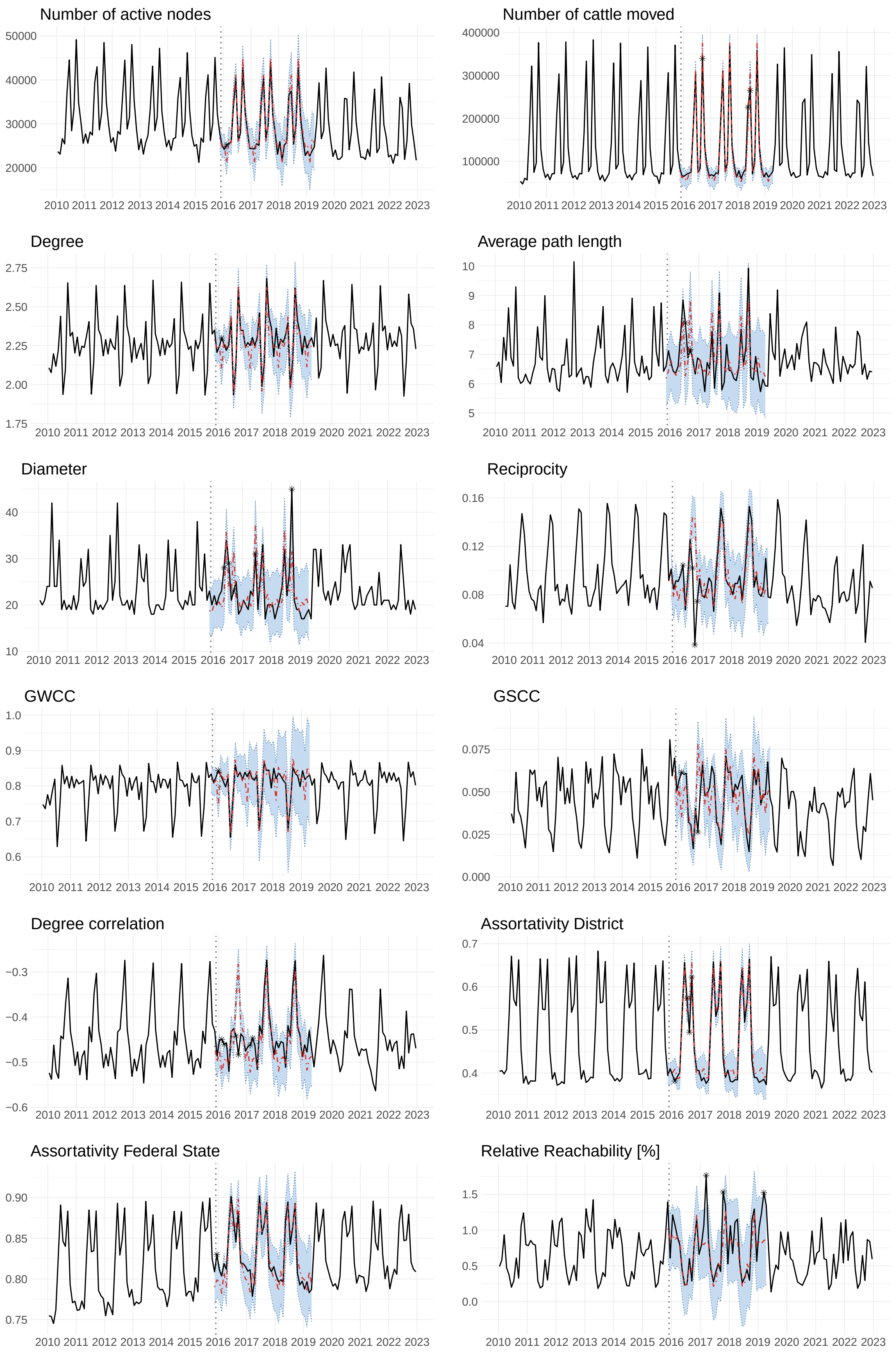

Figure S1. Analysis of cattle movements including movements to pastures before, during, and after the BTV-8 outbreak (2003–2014; left figures) and the BTV-4 outbreak (2010–2022; right figures). Dotted vertical lines indicate the beginning of movement restriction zones. Black lines represent observed data, while red dash-dotted lines show SARIMA predictions with 95% prediction intervals in blue shading. Predictions are shown only during restriction-zone periods; asterisks indicate observations outside the prediction interval.

1.2 Tables

Table S1. Summary of the additional network measures of cattle movements from 2003 to 2022; values shown are yearly medians calculated from monthly networks. For node-level measures — Degree (Total), In/Out-Degree, Betweenness (Directed), Closeness (In), and Closeness (Out) —, the mean values were calculated first.

Metrics	2003	2004	2005	2006	2007	2008	2009	2010	2011	2012
Degree (Total)	2.03	2.07	2.08	2.10	2.11	2.16	2.10	2.15	2.23	2.20
In/Out-Degree	1.05	1.06	1.07	1.08	1.09	1.12	1.09	1.12	1.16	1.14
Reciprocity	0.0584	0.0606	0.0609	0.0621	0.0689	0.0667	0.0714	0.0756	0.0721	0.0725
Transitivity	0.0015	0.0016	0.0015	0.0013	0.0014	0.0013	0.0014	0.0014	0.0014	0.0013
Betweenness (Directed)	9,461	9,377	10,092	11,041	11,219	10,417	9,314	9,221	10,540	10,722
Closeness (In)	0.3647	0.3532	0.3568	0.3469	0.3449	0.3527	0.3432	0.3611	0.3624	0.3742
Closeness (Out)	0.2295	0.2198	0.2136	0.2013	0.1975	0.1949	0.2045	0.2100	0.1937	0.1860

Metrics	2013	2014	2015	2016	2017	2018	2019	2020	2021	2022
Degree (Total)	2.20	2.23	2.22	2.24	2.25	2.23	2.26	2.24	2.24	2.25
In/Out-Degree	1.15	1.17	1.16	1.17	1.18	1.16	1.19	1.16	1.16	1.17
Reciprocity	0.0800	0.0834	0.0833	0.0809	0.0807	0.0814	0.0829	0.0721	0.0765	0.0754
Transitivity	0.0013	0.0012	0.0013	0.0011	0.0010	0.0011	0.0011	0.0012	0.0011	0.0012
Betweenness (Directed)	9,855	11,085	11,894	12,270	11,780	11,515	10,610	10,233	10,717	10,366
Closeness (In)	0.3626	0.3587	0.3651	0.3654	0.3768	0.3723	0.3743	0.3933	0.3895	0.3891
Closeness (Out)	0.2023	0.1920	0.1678	0.1529	0.1373	0.1350	0.1351	0.1419	0.1404	0.1328

Table S2. Summary of the network measures of cattle movements from 2010 to 2022 including movement from and to pastures; values shown are yearly medians calculated from monthly networks. For node-level measures — Degree (Total), In/Out-Degree, Betweenness (Directed), Closeness (In), Closeness (Out), and Relative Reachability —, the mean values were calculated first.

Metrics	2010	2011	2012	2013	2014	2015	2016
Number of active nodes	29,423	29,185	28,818	28,232	27,182	27,344	27,001
Number of batches	37,528	38,235	37,610	36,439	35,622	37,757	34,975
Number of cattle moved	78,024	74,981	75,652	78,811	73,781	78,877	78,936
Number of source-nodes	21,931	22,024	21,598	20,866	20,286	20,935	20,025
Number of target-nodes	13,174	13,085	13,006	12,933	12,540	12,112	12,438
Degree (Total)	2.20	2.28	2.27	2.24	2.27	2.26	2.26
In/Out-Degree	1.15	1.18	1.18	1.18	1.18	1.18	1.18
Average path length	6.67	6.52	6.45	6.41	6.60	6.60	6.86
Diameter	22	21	20	21	20	22	22
GWCC	0.7805	0.8125	0.8196	0.8104	0.8154	0.8109	0.8236
GSCC	0.0382	0.0468	0.0438	0.0474	0.0525	0.0510	0.0485
Degree correlation	-0.4629	-0.4681	-0.4690	-0.4685	-0.4691	-0.4663	-0.4500
Assortativity Federal State	0.7828	0.7826	0.7913	0.8010	0.7975	0.8273	0.8192
Assortativity District	0.4302	0.4249	0.4252	0.4265	0.4284	0.4352	0.4293
Reciprocity	0.0872	0.0859	0.0866	0.0930	0.0928	0.0876	0.0918
Transitivity	0.0016	0.0014	0.0013	0.0014	0.0013	0.0013	0.0012
Betweenness (Directed)	10,252	11,085	10,621	10,185	10,722	11,993	11,838
Closeness (In)	0.3844	0.3767	0.3826	0.3749	0.3674	0.3816	0.3884
Closeness (Out)	0.2240	0.1984	0.1880	0.2017	0.1976	0.1774	0.1553
Relative Reachability [%]	0.5364	0.7132	0.6366	0.8567	0.6726	0.6244	0.7290

Metrics	2017	2018	2019	2020	2021	2022
Number of active nodes	27,204	26,240	26,414	25,091	24,337	24,517
Number of batches	36,655	36,625	35,084	34,914	33,129	34,055
Number of cattle moved	81,474	81,935	87,374	79,370	77,988	80,981
Number of source-nodes	20,460	20,213	19,746	19,107	18,031	18,357
Number of target-nodes	12,163	11,536	12,129	11,487	11,078	10,846
Degree (Total)	2.28	2.31	2.28	2.29	2.25	2.30
In/Out-Degree	1.18	1.20	1.20	1.19	1.17	1.20
Average path length	6.61	6.48	6.51	6.79	6.66	6.60
Diameter	20	21	22	23	21	20
GWCC	0.8279	0.8239	0.8226	0.8126	0.8151	0.8200
GSCC	0.0506	0.0528	0.0475	0.0386	0.0403	0.0440
Degree correlation	-0.4429	-0.4481	-0.4516	-0.4582	-0.4742	-0.4641
Assortativity Federal State	0.8146	0.8057	0.8161	0.8122	0.8228	0.8162
Assortativity District	0.4202	0.4102	0.4276	0.4305	0.4400	0.4319
Reciprocity	0.0918	0.0922	0.0936	0.0788	0.0756	0.0809
Transitivity	0.0011	0.0012	0.0012	0.0013	0.0012	0.0013
Betweenness (Directed)	12,404	12,395	11,045	11,293	10,404	9,598
Closeness (In)	0.3852	0.3862	0.3885	0.4223	0.4137	0.4144
Closeness (Out)	0.1386	0.1415	0.1390	0.1529	0.1538	0.1389
Relative Reachability [%]	0.6625	0.6176	0.7523	0.4695	0.5961	0.6188

Table S3. Loyalty: cattle movement relationships of cattle holdings throughout the years [percentage]. The loyalty measure was calculated for time windows of calendar years. E.g., 3.4 % of cattle holdings having the same movement relationships in 2022 as in 2003.

	2004	2005	2006	2007	2008	2009	2010	2011	2012	2013	2014	2015	2016	2017	2018	2019	2020	2021	2022
2003	17.7	15.0	13.2	11.9	10.6	9.4	8.1	7.5	6.8	6.4	5.9	5.7	5.4	5.1	4.7	4.3	3.9	3.6	3.4
2004		18.5	15.6	13.7	12.0	10.6	9.1	8.4	7.6	7.2	6.5	6.2	5.8	5.4	5.0	4.6	4.1	3.9	3.7
2005			19.8	16.9	14.5	12.7	10.8	9.9	8.9	8.3	7.4	7.2	6.8	6.2	5.8	5.3	4.8	4.5	4.3
2006				20.6	17.1	14.6	12.1	11.0	9.9	9.2	8.3	7.9	7.3	6.8	6.3	5.7	5.2	4.8	4.6
2007					20.5	17.0	13.8	12.4	10.9	10.1	9.0	8.5	7.9	7.2	6.8	6.2	5.6	5.2	4.9
2008						20.7	16.3	14.3	12.5	11.5	10.2	9.5	8.8	8.0	7.5	6.8	6.1	5.7	5.4
2009							20.0	16.9	14.5	13.2	11.6	10.7	9.8	9.0	8.4	7.6	6.8	6.4	6.0
2010								21.8	18.2	16.0	14.0	12.8	11.6	10.7	9.9	9.0	8.1	7.6	7.1
2011									22.1	18.6	15.9	14.3	12.8	11.7	10.8	9.8	8.9	8.3	7.8
2012										22.2	18.3	15.9	14.0	12.9	11.9	10.8	9.7	9.0	8.4
2013											22.3	18.7	16.0	14.3	13.1	11.8	10.6	9.7	9.1
2014												22.5	18.8	16.2	14.8	13.2	11.8	10.8	10.1
2015													24.6	20.3	18.2	16.1	14.2	12.9	11.9
2016														25.1	21.6	18.8	16.4	14.8	13.7
2017															26.4	22.2	19.0	17.0	15.5
2018																26.8	22.3	19.4	17.5
2019																	27.0	22.8	20.2
2020																		26.9	22.8
2021																			27.2
